# Apolipoprotein E and undercaboxylated osteocalcin are associated with bone fragility but not with bone mineral density in osteoarthritis patients

**DOI:** 10.1186/1479-5876-9-S2-P54

**Published:** 2011-11-23

**Authors:** Ana Rodrigues, Joana Caetano-Lopes, Ana Lopes, Ana C Vale, Inês Aleixo, Inês P Perpétuo, Ana S Pena, Alexandra Faustino, Alexandre Sepriano, Joaquim Polido-Pereira, Elsa Vieira-Sousa, Bruno Vidal, José C Romeu, Pedro M Amaral, Luís G Rosa, José A Pereira da Silva, Jacinto Monteiro, Maria F Vaz, João E Fonseca, Helena Canhão

**Affiliations:** 1 Rheumatology Research Unit, Instituto de Medicina Molecular, Faculdade de Medicina da Universidade de Lisboa, Lisbon, Portugal; 2 Rheumatology and Bone Metabolic Diseases Dept., Hospital de Santa Maria, Lisbon, Portugal; 3 Dept. of Mechanical Engineering, Instituto Superior Técnico, Lisbon, Portugal; 4 Ortopaedics Dept., Hospital de Santa Maria, Lisbon, Portugal

## Background

Apolipoprotein E (apoE) gene polymorphisms and undercaboxylated osteocalcin (ucOC) and vitamin K have been associated with fragility fractures and low BMD in general population. The aim of this work was to study whether the effect of apoE gene polymorphisms, seric apoE levels and ucOC influence trabecular bone biomechanics and bone mineral density (BMD) in patients submitted to hip replacement surgery due to advanced primary osteoarthritis.

## Materials and methods

Patients were evaluated for established clinical risk factors (CRFs) for fracture. A dual X-ray absorptiometry (DXA) was performed. ApoE genotyping was performed at positions rs429358 and rs7412. Fasting blood samples were collected at the time of surgery to assess the following parameters: seric apoE, ucOC, Vitamin K, LDL cholesterol, triglycerides and bone turnover markers.

Femoral epiphyses were collected and trabecular bone cylinders were drilled in order to perform compression mechanical tests and analyze bone strength and stiffness.

## Results

Forty-four patients were studied (median 70 years of age, 55% women and 90% postmenopausal, BMI of 27.2 Kg/m2). 6.8% had prevalent fragility fractures, 36% had normal BMD and 64% were osteopenic. The apoE genotype distribution was in accordance with Hardy-Weinberg equilibrium and the E4 allele, previously documented as the risk allele, was present in 16% of the patients. This allele was significantly associated with lower trabecular strength (p=0.004) and stiffness (p =0.07), adjusted for age and gender, but not with BMD. Moreover, E4 allele was associated with higher levels of markers of bone formation: ALP (p=0.067), BSALP (p=0.085) and P1NP (p=0.062). We also found that ucOC was negatively correlated with strength (p=0.077) and stiffness (p=0.047), regardless of patients gender, age or vitamin K, but not with BMD. ApoE levels were also associated with strength (p=0.056; R=-0.322) and stiffness (p=0.001; R=-0.513).

## Conclusions

Other studies have shown that advanced hip osteoarthritis can be associated with a higher BMD, but is not a protective factor for fragility fractures. Our observations suggest that in osteoarthritis patients Apo E4 allele, seric apo E and ucOC are biologic risk factors for fragility in a more independent way from BMD than in the general population.

